# Muskelschmerzen bei Myositis bei einer jungen Patientin – was steckt dahinter?

**DOI:** 10.1007/s00108-024-01761-9

**Published:** 2024-08-09

**Authors:** N. Doblinger, J. Doenz, H. C. Tews, C. Demirci, S. Schmid, B. Michels, M. Müller, A. Kandulski

**Affiliations:** 1 Klinik und Poliklinik für Innere Medizin 1, Gastroenterologie, Hepatologie, Endokrinologie, Rheumatologie und Infektiologie, Universitätsklinikum Regensburg, Franz-Josef-Strauß-Allee 11, 93053 Regensburg, Deutschland; 2 Abteilung für Innere Medizin, Gastroenterologie, St. Theresien-Krankenhaus Nürnberg gGmbH, Nürnberg, Deutschland

**Keywords:** „Early-onset“ kolorektales Karzinom, Paraneoplastisches Syndrom, Vorsorge, Polypektomie, Kollagenose, Early-onset colorectal carcinoma, Paraneoplastic syndrome, Preventive medicine, Polypectomy, Connective tissue disease

## Abstract

Die Myositis ist eine Erkrankung aus dem Formenkreis der Kollagenosen, die am häufigsten bei Frauen zwischen dem 40. und 60. Lebensjahr auftritt. Bei einer deutlichen Assoziation mit malignen Grunderkrankungen wird bei der Erstdiagnose stets ein allgemeines Tumorscreening empfohlen. Das kolorektale Karzinom (KRK) ist eines der häufigsten soliden Malignome des Gastrointestinaltrakts. Typische Risikopopulationen umfassen vor allem Patient*innen jenseits des 55. Lebensjahrs. Die Inzidenz des sogenannten „early-onset“ kolorektalen Karzinoms (EO-KRK) hat in den letzten Jahren signifikant zugenommen. Dabei erkranken zunehmend häufiger Patient*innen im jungen Alter von 20 bis 50 Jahren. Als Grund für diesen Inzidenzanstieg wird eine Zunahme klassischer Risikofaktoren bereits in jüngeren Jahren diskutiert. Wir berichten über eine 34-jährige Patientin, die sich mit dem paraneoplastischen Leitsymptom einer Myositis vorstellte und bei der ursächlich eine sporadische Form eines fortgeschritten metastasierten KRK diagnostiziert wurde. Eine Kontrolle bekannter Risikofaktoren bereits im jungen Erwachsenenalter sowie eine erhöhte Aufmerksamkeit bei Symptompräsentation, beispielsweise in Form von gastrointestinalen Hämorrhagien und paraneoplastischen Symptomen, sind notwendig, um eine rasche Diagnosestellung und ein besseres onkologisches Outcome erreichen zu können.

## Abstract (en)

Myositis is a connective tissue disease which is most frequently diagnosed in women aged 40–60 years. Due to a clear association with underlying malignant diseases, general tumor screening is recommended whenever it is diagnosed. Colorectal carcinoma (CRC) is a common malignant disease, and the typical at-risk group comprised, to date, patients older than 55 years. However, with the rising incidence of so-called early-onset colorectal carcinoma (EO-CRC), an increasingly important patient population is emerging in the 20- to 50-year age range. One reason for the rising incidence is suggested to be an increase in classic risk factors at younger ages. Here, the case of a 34-year-old female patient who presented with the leading paraneoplastic syndrome of myositis and was diagnosed with a sporadic form of CRC is reported. Monitoring of known risk factors as early on as in young adulthood and greater attention in the presence of symptoms such as gastrointestinal hemorrhage or paraneoplastic syndromes seem necessary to compensate for the time delay in diagnosis that currently still exists and the associated worse oncologic outcome.

## Einleitung

Die Myositis wird dem Formenkreis der Kollagenosen zugeordnet und ist durch Inflammation, Schwäche und Schmerzen der Muskulatur charakterisiert. Als eigenständige Erkrankung tritt sie vor allem bei Frauen zwischen dem 40. und 60. Lebensjahr auf [1, 2]. Laborchemisch ist vor allem eine Erhöhung der Kreatinkinase (CK) wegweisend, die bis zum 50fachen des Normwerts ansteigen kann [2]. Insbesondere für die Dermatomyositis besteht eine häufige Assoziation mit malignen Grunderkrankungen (standardisiertes Inzidenzverhältnis von 4,66 für die Dermatomyositis im ersten Jahr nach Diagnosestellung und 1,75 für die Polymyositis; [1]). In der Literatur wird die Erkrankungsrate mit einem Malignom bei Vorliegen verschiedener Formen der Myositis mit 12,1 % angegeben [1, 3], entsprechend wird begrifflich die „cancer-associated myositis“ (CAM) definiert. Besonders häufig ist die Assoziation der CAM mit Tumoren der Ovarien, der Lunge, des Pankreas, des Magens sowie mit kolorektalen Karzinomen und Non-Hodgkin-Lymphomen [4, 5].

Das kolorektale Karzinom (KRK) ist der häufigste maligne Tumor des Gastrointestinaltrakts und stellt weltweit die dritthäufigste Tumorentität mit der zweithäufigsten Ursache für tumorassoziierte Todesfälle dar [6, 7]. Etwa ein Fünftel der Patient*innen wird bereits in einem metastasierten Stadium erstdiagnostiziert, ein weiteres Viertel aller Patient*innen entwickelt im Verlauf der Tumorerkrankung Fernmetastasen [7]. Bei 90 % der Patient*innen wird die Erstdiagnose jenseits des 55. Lebensjahrs gestellt [8]. Durch die Einführung systematischer, populationsbasierter Screeningprogramme konnte die Inzidenz in dieser Altersgruppe deutlich gesenkt werden [9, 10]. Im Gegensatz dazu konnte jedoch in der Gruppe der jüngeren Patient*innen in den letzten Jahren ein Anstieg der Inzidenz beobachtet werden [10]. Diese als „early-onset“ kolorektales Karzinom (EO-KRK) bezeichnete Entität betrifft bereits Patient*innen in einem jungen Alter von 20 bis 50 Jahren. Diese Tumoren werden häufig in einem fortgeschrittenen Stadium erstdiagnostiziert und sind mit schlechter Differenzierung und synchroner Metastasierung (*p* < 0,001) assoziiert [10, 11]. Die Datenlage bezüglich der Prognose ist vergleichsweise dünn und zum Teil widersprüchlich, eine schlechtere Prognose aufgrund der fortgeschritten diagnostizierten Karzinome mit schlechtem Grading wird jedoch angenommen [10].

## Fallbericht

Wir berichten über eine 34-jährige Patientin, die sich initial mit seit 3 Monaten bestehendem Brennen in beiden Oberschenkeln vorstellte. Des Weiteren beklagte die Patientin Dyspnoe, Fatigue, Episoden von Nachtschweiß und Symptome eines Raynaud-Syndroms sowie einer Livedo reticularis im Bereich des Sakralbereichs. Seit 4 Wochen bestünden außerdem intermittierende Episoden von Diarrhö, einmalig sei Blut im Stuhl aufgefallen. Gewicht habe die Patientin nicht verloren, ebenso sei kein Fieber aufgetreten. Es bestanden keine Vorerkrankungen, mit Ausnahme einer Adipositas bei einem BMI von 30,8 kg/m^2^. Eine Dauermedikation wurde ebenso wie Auffälligkeiten in der Familienanamnese oder zurückliegende Infekte verneint.

Laborchemisch zeigten sich ein erhöhtes C‑reaktives Protein sowie eine Leukozytose und eine ausgeprägte Erhöhung der Muskelenzyme CK, CK-MB sowie LDH. Kurz vorausgehende schwere körperliche Anstrengungen und Krampfanfälle sowie medikamenteninduzierte Myopathien wurden anamnestisch ausgeschlossen. Aufgrund der typischen Symptomatik mit Myopathie und Raynaud-Syndrom sowie der starken Erhöhung der Muskelenzyme erschien die Diagnose einer Myositis mit kardialer Beteiligung wahrscheinlicher als beispielsweise ein akuter Myokardinfarkt oder eine Myokarditis, die bei der Laborkonstellation als Differenzialdiagnose zu Bedenken gewesen wären. Antinukleäre Antikörper (ANA) waren entsprechend mit 1:640 erhöht, außerdem waren Ro-52-Antikörper nachweisbar. Eine Echokardiographie und eine kardiale Magnetresonanztomographie (MRT) ergaben einen altersentsprechenden Normalbefund. Eine Kapillarmikroskopie bestätigte die Diagnose eines Raynaud-Syndroms und eine Lungenfunktionsuntersuchung dokumentierte eine mittelschwere restriktive Ventilationsstörung. Hinweise für das Vorliegen einer anderen Kollagenose im Sinne eines Overlap-Syndroms fanden sich klinisch und laborchemisch nicht.

In Zusammenschau der Befunde wurde eine Myositis diagnostiziert und es erfolgte ein Tumorscreening mittels Abdomensonographie und Kontrastmittel-Computertomographie (CT) des Thorax. Bei multiplen echoinhomogenen, malignomsuspekten Leberläsionen wurden zur weiteren differenzialdiagnostischen Abklärung eine MRT der Leber und eine kontrastmittelgestützte Sonographie ergänzt.

**Abb. 1:**
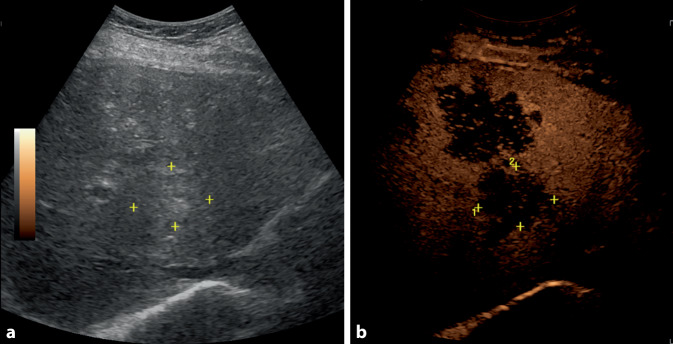
**a** B-Bild-Ultraschall der Leber mit beispielhafter Markierung einer Metastase durch *gelbe* *Kreuze*. **b** Ähnliche Ultraschalleinstellung wie *links* im „contrast-enhanced ultrasound“ (CEUS), man sieht die für Lebermetastasen typische Hypokontrastierung in der Spätphase

In der CT des Thorax imponierten bilaterale, malignomsuspekte pulmonale Filiae. In der kontrastmittelgestützten Sonographie ließen sich multiple, disseminierte malignomsuspekte Läsionen darstellen, welche typischerweise in der venösen Phase eine deutlich verminderte Kontrastmittelspeicherung aufwiesen (Abb. 1).

**Abb. 2:**
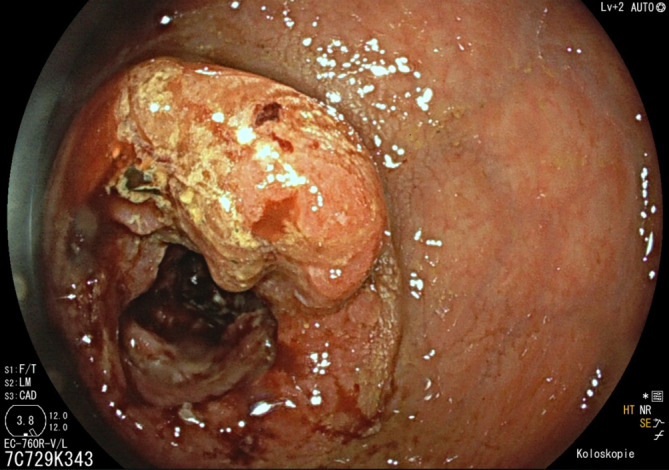
Makroskopisch malignomsuspekte Läsion im Rektum in der Koloskopie

**Abb. 3:**
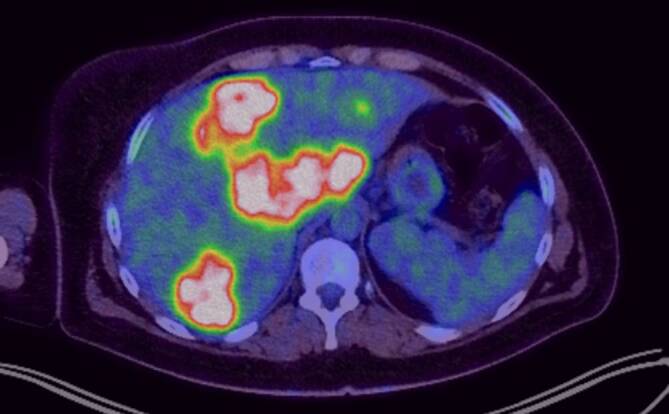
Onkologie-PET mit Low-dose-CT im Sagittalschnitt durch die Leber: deutliche Nuklidmehrspeicherung bei hepatischer Aussaat des KRK

Zur Primariussuche erfolgten bei stattgehabter Hämatochezie eine Gastro- und Koloskopie, wobei sich eine makroskopisch malignomsuspekte Läsion im Rektum zeigte (Abb. 2). In der feingeweblichen Aufarbeitung und der molekularen Analyse bestätigte sich ein mäßiggradig differenziertes Adenokarzinom (G2) mit Mikrosatellitenstabilität (MSS) und Wildtypsequenz hinsichtlich BRAF, KRAS und NRAS. In einer Positronenemissionstomographie(PET)-CT (Abb. 3) zeigten sich neben der disseminierten bipulmonalen sowie hepatischen Metastasierung zusätzlich osteolytische Knochenmetastasen mit FDG-Anreicherung im Os ilium. Darüber hinaus zeigten sich in der PET ausgedehnte perirektale, mesenteriale sowie parailiakale Lymphknotenmetastasen. Eine Biopsie eines Leberherds bestätigte das Vorliegen einer Metastase eines Adenokarzinoms.

Zusammenfassend wurde die Diagnose eines Adenokarzinoms des Rektums im UICC-Stadium IV (G2, MSS, BRAF/KRAS/NRAS-Wildtyp) sowie einer malignomassoziierten Myositis mit myokardialer Beteiligung gestellt.

Zur Behandlung der Myositis erfolgte die Einleitung einer Steroidtherapie. Überlappend wurde eine systemische Chemotherapie zur Behandlung des metastasierten Rektumkarzinoms begonnen. Aufgrund des jungen Patientenalters und bei fehlenden molekularen Markern für eine zielgerichtete oder Immuntherapie wurde eine Therapie mittels FOLFOXIRI und Panitumumab initiiert. 5‑FU wurde bei myokardialer Beteiligung zunächst in 75 %iger Dosisreduktion verabreicht. Zur Kardioprotektion wurde eine niedrig dosierte ACE-Hemmer- und Betablockertherapie initiiert.

## Diskussion

Unser Fallbericht zeigt ein eindrückliches Beispiel eines fortgeschritten metastasierten EO-KRK bei einer 34-jährigen Patientin ohne relevante Risikofaktoren in der Familienanamnese. Die Erstsymptomatik manifestierte sich bei diesem Fall mit einer paraneoplastischen Myositis.

Das EO-KRK hat in den letzten Jahren eine steigende Inzidenz zu verzeichnen und wird epidemiologisch in der Zukunft zunehmend an Bedeutung gewinnen. Zaborowski et al. postulieren, dass im Jahr 2030 bereits 1 von 10 Kolonkarzinomen und 1 von 4 Rektumkarzinomen auf die Altersgruppe unter 50 entfallen wird [10]. Die Gründe hierfür sind noch nicht abschließend geklärt, es liegt jedoch nahe, dass eine Zunahme von individuellen Risikofaktoren wie Adipositas und körperlicher Inaktivität, einer westlichen Diät mit einem hohen Anteil von rotem Fleisch und gesättigten Fettsäuren, Alkohol- und Nikotinabusus sowie Veränderungen im Mikrobiom dafür Ursache tragen [10]. Vor allem ein erhöhtes Körpergewicht ist mit einer chronischen Entzündungsreaktion assoziiert, die die Entstehung verschiedener Krebsformen begünstigt [10]. In einer prospektiven Kohortenstudie mit über 85.000 Teilnehmerinnen konnte gezeigt werden, dass adipöse Frauen ein nahezu doppelt so hohes Risiko tragen, an einem EO-KRK zu erkranken, als Frauen mit einem normalen BMI [12]. Zusätzlich liegen in der Gruppe der EO-KRK im Vergleich zur Gesamtpopulation mit kolorektalem Karzinom vermehrt hereditäre Syndrome wie z. B. das Lynch- oder Polyposis-Syndrom vor. Diese allein erklären jedoch nicht den beobachteten Inzidenzanstieg [10]. Analog dazu präsentierte sich die Patientin in unserem Fallbericht mit einem BMI von über 30 kg/m^2^ und histopathologisch sowie anamnestisch ohne Anhaltspunkt für das Vorliegen eines hereditären Syndroms.

Eine Herausforderung ergibt sich aus der notwendigen Änderung der bisherigen Screeningstrategie in Bezug auf kolorektale Karzinome. Verglichen mit dem klassischen Screeningkollektiv besteht für jüngere Patient*innen momentan noch eine deutliche Verzögerung in der Zeit bis zur Diagnosestellung. So war beispielsweise die mediane Zeit vom Symptombeginn bis zur Diagnosestellung in einer Studie mit 1514 KRK-Patient*innen für solche unter 50 Jahren mehr als 7fach verlängert [13]. Ein bloßes Senken des Screeningalters scheint jedoch keine obligate Strategie zu sein, um diese diagnostische Lücke zu schließen. Stattdessen sollte nach Meinung der Autoren bei typischer Symptomatik im jungen Alter, insbesondere bei Vorliegen einer Adipositas, niedrigschwellig eine Koloskopie angeboten werden (auch bei fehlender Familienanamnese).

Eine erhöhte Aufmerksamkeit sollte generell bei Auftreten typischer paraneoplastischer Syndrome wie der beschriebenen Myositis herrschen. Von den Unterformen der Myositis ist die Dermatomyositis am häufigsten mit Malignomen assoziiert [4, 14]. Daneben spielt die Präsenz verschiedener Autoantikörper eine wichtige Rolle in der Risikostratifizierung. Beispielsweise steigt das individuelle Risiko für eine Krebserkrankung bei einer Dermatomyositis um das 27fache beim Nachweis von Anti-TIF1y-Antikörpern [14]. Weitere Risikofaktoren sind ein höheres Alter bei Diagnosestellung, männliches Geschlecht, das Auftreten von kutanen Nekrosen und Ulzerationen sowie Dysphagie und ein schwerer Krankheitsverlauf [3]. Protektive Faktoren hingegen scheinen eine begleitende interstitielle Lungenerkrankung, ein Raynaud-Syndrom und das Vorhandensein einer Arthralgie bzw. Arthritis zu sein [3]. Eine geeignete Bildgebung bestehend aus CT von Thorax und Abdomen, Ultraschall sowie ggf. Mammographie wird jedoch unabhängig von den Laborwerten und der sonstigen Symptompräsenz als allgemeine Screeningstrategie empfohlen, alternativ wird auch eine PET-CT empfohlen [4, 14].

## Fazit für die Praxis

Der von uns beschriebene Fall stellt ein eindrucksvolles Beispiel für ein sporadisches kolorektales Karzinom im jungen Patientenalter („early-onset“ KRK) sowie das Auftreten einer Myositis als paraneoplastisches Syndrom dar. Eine erhöhte Aufmerksamkeit bei Symptompräsenz sowie ein niedrigschwelliges Angebot zur weiteren diagnostischen Endoskopie insbesondere bei adipösen Patient*innen sind derzeitig die einzigen Strategien, um der steigenden Inzidenz des EO-KRK Rechnung zu tragen.
